# Long-term performance and durability of lime-stabilized oil-contaminated soils

**DOI:** 10.1016/j.heliyon.2025.e41764

**Published:** 2025-01-08

**Authors:** Hadis Nasiri, Navid Khayat, Ahad Nazarpour

**Affiliations:** a Department of Civil Engineering, Ahvaz Branch, Islamic Azad University, Ahvaz, Iran; b Department of Geology, Ahvaz Branch, Islamic Azad University, Ahvaz, Iran

**Keywords:** Microstructure, Unconfined compressive strength, Wet-dry cycles, Oil-contaminated soil, Mechanical property, Scanning electron microscopy

## Abstract

In oil-rich regions, the increasing risk of oil spills on soil is largely attributed to intensified extraction and transportation activities. This situation necessitates a focus on the short-term and long-term strength of contaminated soils. While existing literature primarily evaluates the oil-contaminated soils over short-term periods, typically up to 28 days, it is essential to investigate their long-term performance, extending the evaluation period to 365 days. This study addresses the critical gap in understanding the long-term performance of soils contaminated with 4 %, 7 %, and 10 % oil by evaluating the effectiveness of lime stabilization over a one-year period. Laboratory tests were conducted on soils treated with varying lime contents (0 %, 3 %, 6 %, and 9 %) and cured for 1, 14, 28, and 365 days. Key performance indicators, including unconfined compressive strength (UCS), California Bearing Ratio (CBR), and durability under wet-dry cycles, were measured. The results demonstrate that a 6 % lime content significantly improves long-term UCS, with strength gains ranging from 16.6 % to 24.5 % while enhancing resilience to wet-dry cycles. Microstructural analyses confirmed the formation of calcium-aluminum-silicate-hydrate (C-A-S-H) phases, contributing to the observed strength and durability improvements. This research underscores the potential of lime stabilization as a sustainable solution for managing oil-contaminated soils, reducing reliance on raw materials, and promoting more sustainable infrastructure development.

## 1 Introduction

Geotechnical engineering is crucial in promoting sustainable infrastructure by using recycled materials in earthwork structures [[1], [2], [3]]. Using these materials in ground improvement projects offers significant economic benefits and positive environmental impacts [3,4]. However, the expansion of the chemical industry, particularly after the industrial era, has led to increased levels of pollutants in both the natural and human environment [5]. Among these industries, the petrochemical sector remains dominant in the global energy landscape despite growing investments in and interest in alternative energies. Oil remains a critical energy resource for modern society, and despite stringent safety measures, oil spills remain an inevitable challenge throughout the production, transportation, refining, and consumption of crude oil [6]. A significant number of global regions are affected by oil contamination, necessitating the implementation of remediation strategies [7]. The effects of oil on soils and the serious environmental concerns that arise from it are complex issues that demand greater understanding. Oil spills pose a significant problem in oil-rich regions worldwide [8]. As a result, an increasing number of studies over recent decades have focused on oil contamination in soil due to its rising prevalence.

One potential solution to mitigate the adverse effects of oil-contaminated soils on the environment is to repurpose these soils for construction projects [9]. Geotechnical engineers face substantial costs in disposing of large volumes of soil with unfavorable geotechnical characteristics and in transporting suitable soil from distant locations [10]. Therefore, treating contaminated soils becomes essential. The reuse of treated oil-contaminated soil is advantageous for two primary reasons. First, transporting and disposing of contaminated soil reduces the cost and time. Instead of moving the contaminated soil to a disposal site, on-site treatment through additive mixing and soil stabilization techniques is possible. Second, stabilizing oil-contaminated soils allows for recovering and reusing contaminated soil in construction projects, transforming it into a suitable material for geotechnical applications [[11], [12], [13], [14]]. This approach significantly lowers costs and offers economic benefits in areas affected by oil leakage [15].

Previous studies have primarily focused on the short-term behavior of sand contaminated with crude oil [16,17], as granular materials are more commonly used in geotechnical projects. In contrast, fine-grained oil-contaminated soils are often overlooked. However, fine-grained soils are common in tropical regions like southern and western Iran, and oil contamination of these soils is inevitable. Fine-grained soils are chemically sensitive to pollutants and can retain or absorb contaminants for extended periods. For example, studies on coastal soils in Bushehr, Iran, have revealed contamination from crude oil [18]. Ignoring the issue of oil-contaminated fine-grained soils can lead to substantial costs due to their impact on structural integrity [16]. Therefore, exploring techniques to modify or stabilize these soils for more effective treatment is essential.

Several studies have investigated stabilizing oil-contaminated fine-grained soils, focusing primarily on their short-term strength [[18], [19], [20], [21], [22], [23]]. These studies have shown that contaminated soil can be modified for use in construction projects rather than being disposed of, suggesting the potential for re-utilization [16]. For example, Salimnezhad et al. (2021) observed that oil contamination causes clay samples to become brittle, reducing short-term unconfined compressive strength (UCS) [24]. Oluwatuyi et al. (2020) found that increasing the cement-lime content from 5 % to 15 % increased UCS values for stabilized clay, achieving 350 kPa for uncured samples and 1160 kPa for 28-day (short-term) cured samples [16]. Despite these findings, limited research exists on oil-contaminated clays' long-term (1-year) behavior. Given the importance of soil stability in transportation infrastructure, this research explores the effectiveness of lime stabilization in improving the strength and durability of oil-contaminated soils. Through experimental studies and microstructural analyses, this research evaluates the performance of these soils under environmental stresses, such as wet-dry cycles, providing insights into their long-term behavior.

To date, there is a notable gap in the literature regarding the long-term stabilization of oil-contaminated fine-grained soils, particularly over extended curing periods. This gap is especially evident in tropical regions where oil contamination of fine-grained soils is widespread, yet research on their stabilization is limited to short-term behavior. Thus, the aim of this research is to address this deficiency by (i) evaluating the long-term (1-year) performance of lime stabilization in soils contaminated with varying oil contents (4 %, 7 %, and 10 %), (ii) determining the optimal lime content for achieving improved strength and durability, and (iii) performing microstructural analysis to confirm the formation of stabilization products. The experimental results were assessed through mechanical properties, unconfined compressive strength (UCS), California Bearing Ratio (CBR), and durability tests, offering critical insights into the suitability of lime-stabilized oil-contaminated soils for sustainable construction applications.

## 2 Materials and sample preparation

### 2.1 Properties of clay soil

**Fig. 1:**
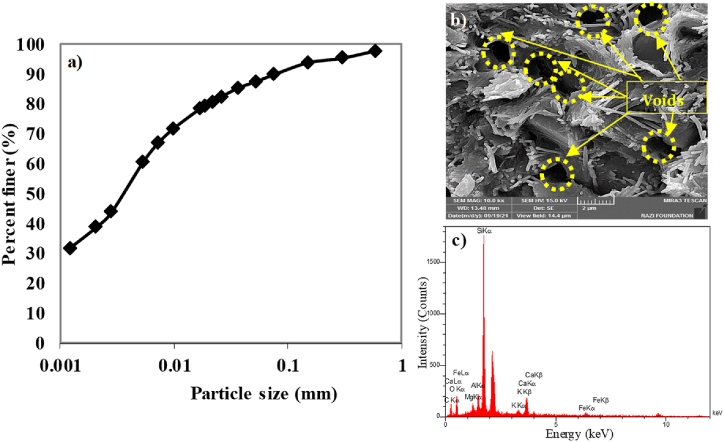
a) Particle size gradation curve b) FE-SEM image c) EDS spectrum of the collected clay soil.

The soil sample used in this study was collected from Ahvaz, Khuzestan, Iran. The soil's plasticity index and liquid limit were measured at 27 % and 54 %, respectively. The particle size distribution curve of the collected soil is shown in Fig. 1a. Fig. 1b presents the voids in the matrix of non-contaminated soil, observed using field-emission scanning electron microscopy (FE-SEM). In contrast, Fig. 1c illustrates the presence of silica, aluminum, iron, calcium, and magnesium, as indicated by the non-contaminated soil's energy-dispersive spectroscopy (EDS) spectrum. According to the Unified Soil Classification System (USCS), the soil sample is classified as *CH* (high-plasticity clay). A summary of the soil's physical characteristics and chemical composition is provided in Table 1. The predominant oxides in the soil are silicon dioxide (SiO₂) and aluminum oxide (Al₂O₃), which account for approximately 66 % of the total mass.

**Table 1:** Physical and chemical properties of investigated clay soil.

**Physical Properties**	**Value**	**Chemical Composition**	**Value (%)**
Gravel (%)	0	SiO_2_	54.78
Sand (%)	10	Al_2_O_3_	11.55
Fines (%)	90	Fe_2_O_3_	2.62
Liquid limit (LL)	54	CaO	9.52
Plastic limit (PL)	28	MgO	3.67
Plasticity index (PI)	26	Na_2_O	1.67
Specific Gravity (G_s_)	2.62	K_2_O	1.22
Soil classification (USCS)	CH	Ti_2_O	0.32
Activity (%)	0.69	MnO	0.04
		L.O.I^a^	14.61

a Loss on ignition.

### 2.2 Properties of contaminant oil

The oil used in this study is desalinated crude oil obtained from the Karun 1 oil and gas field in Ahvaz, Iran. The desalination process removes toxic gases and water from the oil. It has a density of 0.858 g/cm³, is in a semi-liquid state, and has a dark brown color. Its primary chemical components are hydrocarbons, predominantly carbon (C) and hydrogen (H). Several soil samples were collected from oil-contaminated sites in Khuzestan and analyzed using a Soxhlet extractor to determine the oil content. The highest recorded oil content in the contaminated sites was 10 %. Therefore, a 10 % concentration and lower contamination levels (4 % and 7 %) were selected to investigate the behavior of soils with varying oil contamination levels. This allowed for an assessment of the effect of different oil percentages on soil behavior. After obtaining the crude oil and transferring it to the laboratory, the oil was manually blended with the soil, simulating an artificial contamination process.

### 2.3 Properties of lime (LI)

Hydrated lime, with a purity exceeding 72 %, as specified by ASTM C977, was procured from the Khuzestan Ahak Company in Khuzestan, Iran. Table 2 provides the chemical composition of the lime, determined through X-ray fluorescence (XRF) analysis. As expected, the primary component of the lime is calcium oxide (CaO). The lime content used in this study included concentrations of 0 %, 3 %, 6 %, and 9 %, based on recommendations from previous studies [[25], [26], [27]].

**Table 2:** Chemical compositions of lime.

Chemical Composition	Lime
	Value (%)
SiO_2_	0.95
Al_2_O_3_	0.30
Fe_2_O_3_	0.24
CaO	72.04
MgO	0.64
Na_2_O	0.13
K_2_O	0.11
SrO	0.31
P_2_O_5_	0.04
L.O.I^a^	25.24

a Loss on ignition.

### 2.4 Preparing the oil-contaminated soil samples

To ensure proper soil contamination, the literature widely recommends mixing the soil with the required percentage of the contaminant and then storing it in nylon bags for approximately one week to allow for complete absorption of the contaminant by the soil [[28], [29], [30], [31]]. In this study, the clay was contaminated with crude oil at three different concentrations (4 %, 7 %, and 10 % by dry weight of the clay). The dry soil was thoroughly mixed with the contaminant and then stored in the laboratory for one week to ensure complete oil absorption by the soil particles.

### 2.5 Preparing lime-stabilized samples of oil-contaminated soil

Initially, the oil-contaminated soil was mixed with varying proportions of lime (0 %, 3 %, 6 %, and 9 % by weight). The mixture was thoroughly combined to ensure homogeneity. Distilled water was then added to the soil to achieve its optimum moisture content (OMC), and the mixture was blended until a uniform consistency was obtained. The prepared mixture was transferred into nylon bags and stored for 24 h for proper curing. After this period, the samples were ready for further testing and utilization.

## 3 Testing methods

**Fig. 2:**
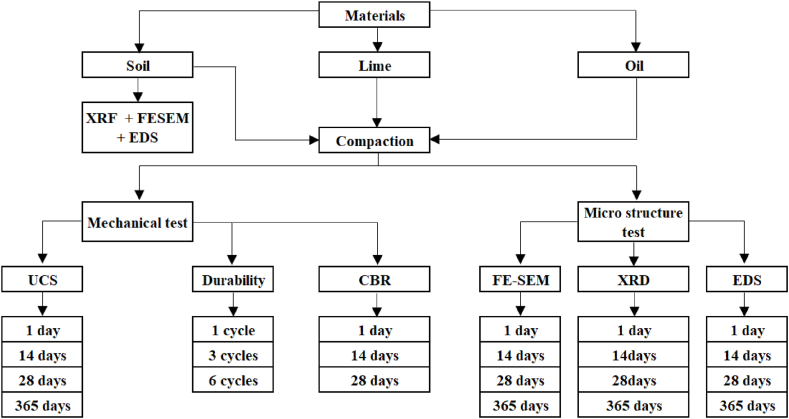
Flow chart illustrating the test methodology adopted in the study.

The test methodology adopted was presented in the flowchart (Fig. 2).

### 3.1 Mechanical tests

The mixing designs for the oil-contaminated soil experiments are presented in Table 3. A total of 264 mechanical tests were conducted, including 144 short-term UCS tests (with 3 repetitions for each test), 36 durability tests, 48 CBR tests, and 36 long-term tests (with 3 repetitions for each test).

**Table 3:** Summary of the testing program.

Oil (%)	Lime (%)	Curing time (day)	Tests^a^
0	0	1, 14, 28, and 365	UCS (Short-term and Long-term), and durability
4	0		
7	0		
10	0		
0	3		
4	3		
7	3		
10	3		
0	6		
4	6		
7	6		
10	6		
0	9		
4	9		
7	9		
10	9		

a Number of short-term UCS tests: 144, number of durability tests: 36, number of CBR tests: 48, and number of long-term tests: 36.

#### 3.1.1 UCS (ASTM D2166)

The samples, prepared using the mixture from the previous step, underwent Proctor soil compaction testing. Subsequently, three cores were extracted from each mold. The compacted samples were cylindrical (76 mm in height and 38 mm in diameter) and composed of soil contaminated with various stabilizers. After each curing period, the samples were subjected to UCS testing, and the resulting strength values were compared to those of non-contaminated samples as a percentage.

Three samples were prepared for each tested percentage of oil-contaminated soil as long-term samples, and the results were averaged. It is important to note that before UCS testing and after one year of curing, the mass of each sample was measured and compared to its initial mass before curing. The measurements indicated minimal and negligible mass changes, signifying no significant moisture loss in the samples after curing.

#### 3.1.2 Durability (ASTM D559)

For the durability tests conducted according to ASTM D559, the samples prepared earlier were immersed in water for 5 h, followed by drying in an oven at 71 ± 3 °C for 42 h. This cycle was repeated for 1, 3, and 6 wet-dry (w-d) cycles. The number of cycles may vary depending on the soil type and road; typically, 1 to 4 cycles are appropriate for silty or clayey soils [32]. This study, however, considered six w-d cycles.

#### 3.1.3 CBR (ASTM D1883)

In CBR, the rammer weight is equal to 4.5 kg, and the free-fall height is equal to 457.2 mm, which is the same as the modified compaction test. The CBR values of the soil sample were calculated as per ASTM D 1883. The soil sample that was stabilized was compacted in a mold at the maximum dry unit weight (MDU) and OMC. Then, the mold was placed with a plastic sheet on top for curing. The samples were tested after curing days.

### 3.2 Morphological and mineralogical studies

A range of techniques, including X-ray diffraction (XRD), SEM, and EDS, were employed to assess the microstructure and morphology of the soil samples. These microstructural analyses were performed with UCS tests to understand how the soil's strength parameters evolved.

## 4 Results and discussion

### 4.1 MDU and OMC results

**Fig. 3:**
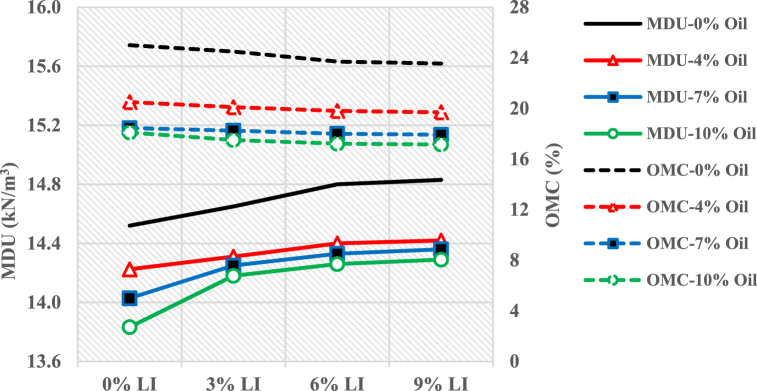
Compaction results for non-contaminated and oil-contaminated soils with of Lime.

Fig. 3 presents the compaction test results, showing the relationship between MDU and OMC versus different lime percentages for both non-contaminated and oil-contaminated samples. Generally, MDU and OMC values decrease as the oil percentage increases in the contaminated soil samples. The presence of oil reduces water absorption and results in energy loss, leading to reduced compaction efficiency [24].

As the oil percentage increases to 10 %, the MDU value decreases to 13.8 kN/m³, compared to 14.5 kN/m³ for non-contaminated soil, a reduction of approximately 4.8 %. This decrease is attributed to oil's higher viscosity than water, which leads to energy loss during compaction [33]. Oil has a lower density than water; thus, when oil occupies the pore space instead of water, it reduces the bulk fluid density, further decreasing the MDU in oil-contaminated samples. Furthermore, oil limits water access to soil particles, wetting only the external surface, thereby reducing compaction efficiency [34].

Adding lime to oil-contaminated soil resulted in an increase in MDU and a decrease in OMC relative to non-contaminated soil. For example, a 2.5 % increase in MDU was observed in samples containing 10 % oil and 3 % lime compared to oil-contaminated samples without lime. Similarly, MDU increased by 3.1 % and 3.3 % in samples with 6 % and 9 % lime, respectively. This is attributed to the higher specific gravity of lime, which increases the soil's overall specific weight [35,36].

As lime content increased, OMC decreased. In the sample containing 10 % oil and 3 % lime, OMC dropped to 17.5 %, representing a 3.3 % decrease compared to a similar oil-contaminated sample without lime. For samples with 6 % and 9 % lime, OMC decreased by 4.9 % and 5.3 %, respectively. The decrease in OMC is due to the chemical interactions between lime and water, which promote the stabilization and strengthening of the mixture. These results also showed no significant differences between samples stabilized with 6 % and 9 % lime in MDU or OMC.

### 4.2 UCS

**Fig. 4:**
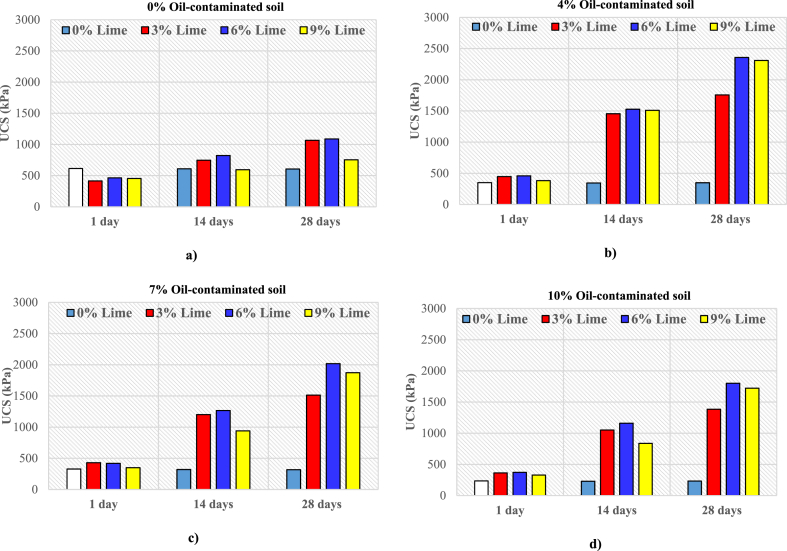
UCS for non-contaminated and oil-contaminated soils with different percent of lime of a) 0 % Oil b) 4 % Oil c) 7 % Oil d) 10 % Oil.

Fig. 4 presents the UCS results for oil-contaminated soil samples with varying lime contents, evaluated at 1, 14, and 28 days of treatment. The comparison of UCS values for soils contaminated by various oil percentages revealed that oil altered the soil structure. The UCS of oil-contaminated soil decreased significantly as the oil percentage increased from 4 % to 10 %. This is likely due to oil's higher viscosity than water, which may coat the soil particles, inhibiting particle bonding and reducing soil strength [37]. At 28 days of curing, the UCS was 348 kPa for soil contaminated with 4 % oil but only 233 kPa for soil with 10 % oil at the same curing period.

For soil contaminated with 4 % oil and stabilized with 3 % lime, UCS increased by 27.7 %, 321.7 %, and 404.9 % at 1, 14, and 28 days of curing, respectively (Fig. 4b). As lime content increased from 3 % to 6 %, UCS improved further. At 1 day of curing, the UCS of soil contaminated with 4 % oil and stabilized with 6 % lime reached 460 kPa, rising to 1527 kPa at 14 days and 2357 kPa at 28 days.

The results for soils contaminated with 7 % (Fig. 4c) and 10 % (Fig. 4d) oil and stabilized with varying lime percentages also showed increases in UCS across all curing ages. The highest UCS was recorded for soils contaminated with 7 % and 10 % oil and stabilized with 6 % lime. At 28 days of curing, the UCS of soil contaminated with 7 % oil and stabilized with 6 % lime reached 2018 kPa. In soils with 10 % oil and 6 % lime, UCS increased by 58.1 %, 404.4 %, and 673.0 % at 1, 14, and 28 days of curing, respectively.

This study assessed strength criteria, including the USEPA minimum strength criterion for deeming a material non-hazardous (i.e., 340 kPa after 28 days of curing for roads [38]. The results shown in Fig. 4 demonstrate that non-contaminated soils and soils contaminated with 4 %–10 % oil and stabilized with 3 %–9 % lime meet the strength criterion. Additionally, soils contaminated with 4 % and 7 % oil, even without lime stabilization, satisfy this criterion. However, soils contaminated with 10 % oil without lime do not meet the required UCS for road applications.

To make the results more applicable, UCS and strain energy (SE) values were compared to untreated control samples (Table 4). SE is the energy required to deform a sample to the strain equivalent to peak stress, calculated from the area under the stress-strain curve [39]. Positive SE values indicate increased strength and energy efficiency, while negative values suggest reduced strength and SE compared to control samples, which had the same oil contamination levels but without lime treatment. This comparison provides a clearer insight into lime's impact on stabilizing oil-contaminated soils.

**Table 4:** UCS and SE differences of the soils containing 4 %–10 % of oil and 3 %–9 % of lime from the controlled samples.

Equation	Equation No.	Difference					
		1 day-curing		14 days-curing		28 days-curing	
		UCS (kPa)	SE (kJ/m^3^)	UCS (kPa)	SE (kJ/m^3^)	UCS (kPa)	SE (kJ/m^3^)
y = x (S+4%O+3%L)-x (S+4%O)	1	96.8	−23.28	1110.0	−2.46	1408.5	−5.88
y = x (S+4%O+6%L)-x (S+4%O)	2	110.0	−17.04	1182.0	4.31	2009.0	11.22
y = x (S+4%O+9%L)-x (S+4%O)	3	32.3	−21.47	1165.5	4.76	1961.0	12.92
y = x (S+7%O+3%L)-x (S+7%O)	4	101.2	−12.20	880.9	5.79	1196.0	5.55
y = x (S+7%O+6%L)-x (S+7%O)	5	91.2	−8.36	946.3	7.08	1701.0	28.91
y = x (S+7%O+9%L)-x (S+7%O)	6	22.2	−9.74	619.8	−5.45	1555.0	13.21
y = x (S+10%O+3%L)-x (S+10%O)	7	128.6	−2.66	821.0	8.65	1151.5	21.03
y = x (S+10%O+6%L)-x (S+10%O)	8	137.8	2.74	930.0	15.39	1568.0	25.06
y = x (S+10%O+9%L)-x (S+10%O)	9	93.6	−1.68	608.0	6.10	1490.0	15.02

**Fig. 5:**
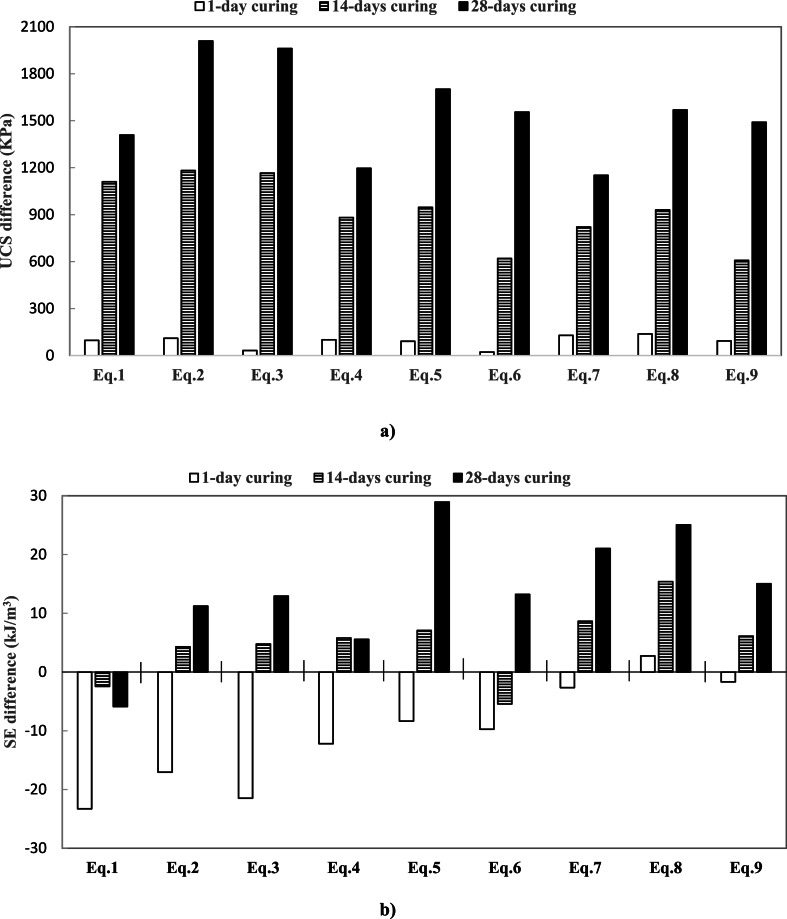
UCS and SE differences of the soils containing 4 %–10 % of oil and 3 %–9 % of lime from the controlled samples.: a) UCS difference of Eqs. (1)–(9); b) SE difference of Eqs. (1) to (9).

Fig. 5 illustrates the differences in UCS and SE for various equations (Eq. (1) to Eq. (9)) across three curing periods: 1 day, 14 days, and 28 days. The data suggests that more extended curing periods generally result in greater UCS increases, with the most significant improvements observed after 28 days of curing. In contrast, SE differences show more varied responses to curing periods. Overall, prolonged curing enhances the mechanical properties of the materials tested, as evidenced by higher UCS and SE values after 28 days.

In Fig. 5a, UCS differences increased significantly with extended curing times across all equations, and the 1-day curing period yielded the lowest UCS differences, typically below 300 kPa. At 14 days, UCS differences increased moderately, generally between 600 kPa and 900 kPa. During the 28-day curing period, they exhibited the highest UCS differences, consistently exceeding 1200 kPa, with equations Eq. (3) and Eq. (6) reaching up to 2100 kPa. Positive UCS differences indicate strength increases due to lime stabilization, with more significant differences observed at extended curing periods, highlighting the progressive effectiveness of lime treatment. This study did not observe negative UCS values, which would indicate a weakening effect.

Fig. 5b reveals a more complex relationship between SE differences and curing periods. After 1 day of curing, most equations exhibited negative SE differences, indicating reduced energy efficiency, except for Eq. (5), which had a slight positive value. By 14 days of curing, some equations, such as Eq. (3) and Eq. (8), displayed positive SE differences, while others, such as Eq. (1) and Eq. (4), remained negative. After 28 days, most equations showed positive SE differences, with Eq. (5) showing the most significant increase, exceeding 20 kJ/m³, followed by Eq. (7) and Eq. (8). Positive SE values suggest improved energy efficiency, indicating that less energy is required for the soil to reach a given level of deformation. This implies that lime stabilization enhances soil strength and energy efficiency under load. Conversely, negative SE values indicate that more energy is required for deformation, which may suggest a trade-off between strength gain and energy efficiency depending on lime content.

### 4.3 Long-term performance

Based on the findings discussed in the Short-term Performance section, the highest UCS in both non-contaminated and contaminated samples (with 4 %, 7 %, and 10 % oil) is achieved by the lime-stabilized mixtures containing 6 % lime, which has been identified as the optimum lime content according to UCS testing. To further investigate the long-term effects of curing time, these samples were subjected to one year (365 days) of curing, followed by UCS testing.

**Fig. 6:**
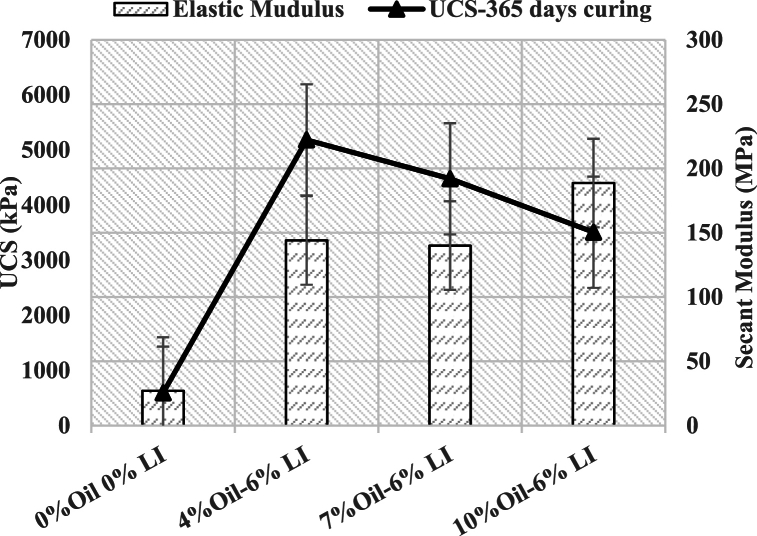
UCS and secant modulus of long-term samples.

Fig. 6 shows the long-term UCS results of stabilized samples at 365 days. At this one-year curing period, a 6 % lime content in soil samples contaminated with 4 %, 7 %, and 10 % oil resulted in a 9-, 8-, and 6-fold increase in UCS, respectively, compared to non-contaminated, unstabilized soil samples cured for the same period. For example, the UCS of samples contaminated with 4 % oil and stabilized with 6 % lime increased 9 times after 365 days of curing compared to a non-contaminated and unstabilized sample at the same curing age. Similarly, samples contaminated with 7 % and 10 % oil and stabilized with 6 % lime showed an 8-fold and 6-fold increase in UCS after 365 days of curing, respectively, compared to the non-contaminated and unstabilized reference sample at 365 days.

The **secant modulus**, which represents the strain corresponding to 50 % of the maximum principal stress difference on the stress-strain curve, was also analyzed. This modulus is a crucial measure of soil stiffness and is calculated by identifying the strain at half of the peak stress. The results indicate that the samples contaminated with 4 % and 7 % oil and stabilized with 6 % lime exhibited similar secant modulus values. However, when the oil content reached 10 %, a notable reduction in secant modulus was observed, indicating a decrease in soil stiffness at higher contamination levels despite the stabilization effect of lime.

### 4.4 Durability

**Fig. 7:**
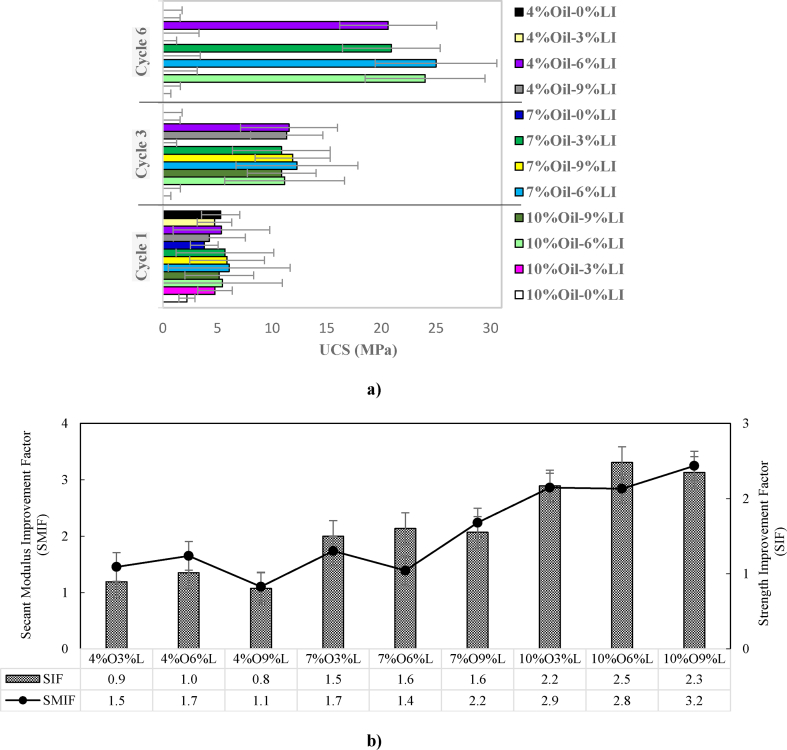
a) UCS for non-contaminated and oil-contaminated soils with different percent of lime b) SIF and SMIF of oil-contaminated and stabilized samples with lime based on the durability test after 1 cycle wet-dry.

Fig. 7a presents the results of durability tests on oil-contaminated and non-contaminated soil samples. All soil samples maintained their entire UCS after one wet-dry cycle. However, when comparing the results of contaminated samples with varying oil percentages, it can be observed that as the oil percentage increases from 7 % to 10 %, the durability of the samples decreases. This decline in durability aligns with the pattern observed in the UCS tests.

In samples containing 4 % oil, the highest UCS was observed in those stabilized with 6 % lime, with strength three times higher than that of samples under normal conditions during the first cycle. In this context, “normal conditions” refer to 28-day samples without wet-dry cycles. In samples with 7 % oil, the UCS stabilized with 3 %, 6 %, and 9 % lime after the first cycle, which was 5.7, 1.6, and 1.9 times higher, respectively, compared to samples with the same oil percentage but without lime stabilization. A similar trend was observed in the 10 % oil samples, where increasing the lime percentage to 6 % resulted in higher UCS values than in unstabilized samples.

In **Cycle 3**, all unstabilized oil-contaminated samples were destroyed. However, when 3 % lime was added, only the samples containing 7 % oil exhibited a UCS that was seven times higher than that of unstabilized samples after wet-dry cycling. When the lime content was increased to 6 %, the oil-contaminated samples displayed durability 5 to 6 times higher than that of samples under normal conditions. Additionally, after the third cycle, the samples containing 4 %, 7 %, and 10 % oil and stabilized with 9 % lime had durability values of 5, 6, and 6, respectively.

In **Cycle 6**, most of the samples disintegrated in water. Among the samples contaminated with 4 % and 10 % oil, those stabilized with 6 % lime exhibited 9 % and 14 % durabilities, respectively. Among the samples contaminated with 7 % oil, those stabilized with 3 % and 6 % lime exhibited UCS values of 20.9 MPa and 25.0 MPa, respectively. The durability test results from Cycles 1, 3, and 6 indicate that as the oil percentage increases, the durability decreases, whereas increasing the lime content to 6 % improves the durability of the samples.

Four non-dimensional parameters were used to evaluate the performance improvement in stabilized samples, including the **Strength Improvement Factor (SIF)** and the **Secant Modulus Improvement Factor (SMIF)**, as defined by Eqs. (1), (2):

(1) $$\text{Strength}\;\text{Improvement}\;\text{Factor}\;\left(\text{SIF}\right)=\frac{\text{UCS}\;\text{of}\;\text{stabilized}\;\text{sample}\;\text{cured}\;\text{at}\;n\;\text{days}}{\text{UCS}\;\text{of}\;\text{unstabilized}\;\text{sample}\;\text{cured}\;\text{at}\;n\;\text{days}\;}$$

(2) $$\text{Secant}\;\text{Modulus}\;\text{Improvement}\;\text{Factor}\;\left(\text{SMIF}\right)=\frac{\text{Secant}\;\text{Modulus}\;\text{of}\;\text{stabilized}\;\text{sample}\;\text{cured}\;\text{at}\;n\;\text{days}}{\text{Secant}\;\text{Modulus}\;\text{of}\;\text{unstabilized}\;\text{sample}\;\text{cured}\;\text{at}\;n\;\text{days}\;}$$

In Fig. 7b, the graph illustrates the SMIF and SIF values for various percentages of lime. The graph shows that increasing the lime content improves the SMIF and SIF.

• The **SIF** values exhibit an increasing trend with higher oil percentages. For mixtures with 4 % oil, SIF values are below 1, indicating lower strength improvement. In contrast, the mixtures with 7 % oil show SIF values ranging from 1.5 to 2.2, indicating moderate strength improvement. The highest SIF value of 2.9 was observed in the mixture containing 10 % oil and 3 % lime. Other mixtures with 10 % oil also exhibited high SIF values, indicating significant strength improvement due to lime stabilization.
• The **SMIF** values also show an upward trend with increasing oil content. For the 4 % oil mixtures, the SMIF values range from 1.1 to 1.7, indicating a modest improvement in soil stiffness. As the oil content increased to 7 %, the SMIF values fluctuated between 1.4 and 2.2. The highest SMIF value of 3.2 was achieved in the mixture containing 10 % oil and 9 % lime, demonstrating a significant increase in soil stiffness and deformation resistance with higher oil and lime content.

These findings underscore the positive effect of lime stabilization, particularly at a lime content of 6 %, in enhancing both the durability and mechanical properties of oil-contaminated soils.

### 4.5 CBR test

**Fig. 8:**
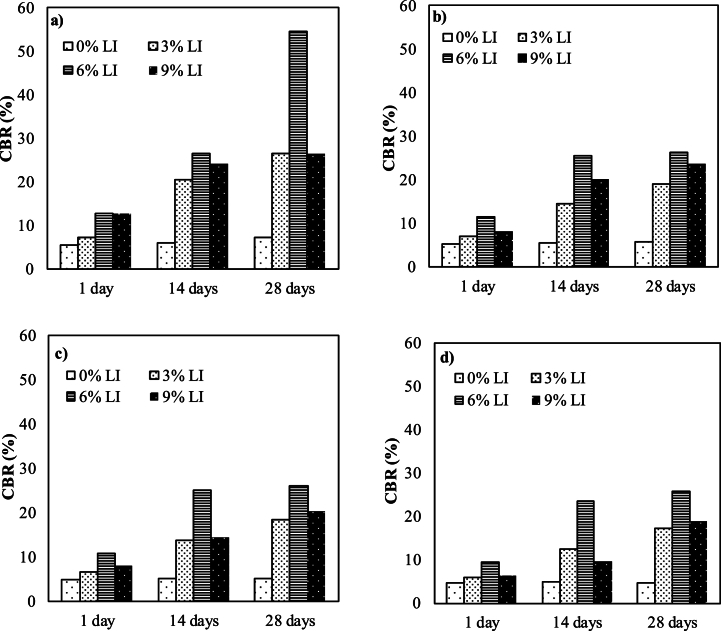
CBR for non-contaminated and oil-contaminated soils with different percent of lime of a) 0 % Oil b) 4 % Oil c) 7 % Oil d) 10 % Oil.

CBR tests were conducted to analyze the effect of oil-contaminated soil with and without lime on clay's California Bearing Ratio (CBR). Fig. 8 presents the CBR results of lime-treated clay with various oil contents. As the lime content increased, the CBR of the oil-contaminated soil also increased. This indicates that lime improves the load-bearing capacity of the clay. Adding lime enhanced the CBR of lime-treated clay, with the maximum gain observed at 6 % lime content.

The maximum CBR gains observed for 6 % lime content in soils contaminated with 4 %, 7 %, and 10 % oil (Fig. 8b, c, and 8d) were 358 %, 412 %, and 454 % higher than those of non-stabilized clay after a curing period of 28 days. For the same curing period, the CBR values for 9 % lime-treated clay contaminated with 4 %, 7 %, and 10 % oil were 23.7 %, 20.4 %, and 18.9 %, respectively. These values were 4.10, 4.01, and 4.06 times higher than those of non-stabilized clay.

Although CBR increased with the addition of 9 % lime, the gains were lower than those observed with 6 % lime. This reduction in performance is attributed to the decrease in MDU weight and the increase in OMC of the clay when the lime content increased from 6 % to 9 % [40]. The contribution of lime is significant in improving the CBR of clay, particularly over the 28-day curing period, due to pozzolanic reactions between lime and clay that are more active as the curing process progresses.

### 4.6 Micro-structure and composition

#### 4.6.1 XRD

**Fig. 9:**
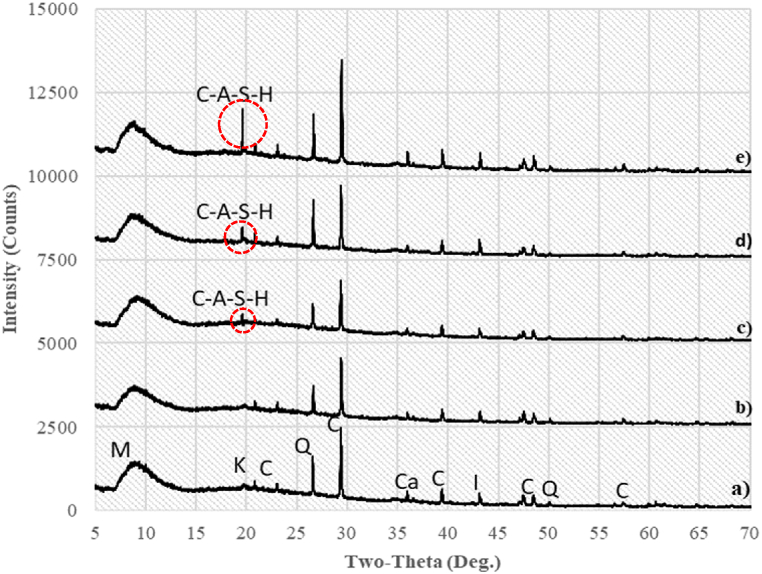
XRD intensity counts for soil with a) 10%O 0%L and 28-d curing b) 10%O 6 % L and 1-d curing c) 10%O 6 % L and 14-d curing d) 10%O 6 % L and 28-d curing e) 10%O 6 % L and 365-d curing [C: calcite, Q: quartz, I: ilitte; M: montmorillonite, Ca: Calcium silicate, K: kaolinite, C-A-S-H: Calcium aluminium silicate hydrate].

The **XRD** results for 10 % oil-contaminated soil stabilized with 6 % lime after 1, 14, 28, and 365 days of curing are shown in Fig. 9. The mineralogical composition of both unstabilized and stabilized soil was nearly identical and consisted primarily of **kaolinite (K)**, **quartz (Q)**, **calcite (C)**, **calcium silicate (Ca)**, and **montmorillonite (M)** as the dominant mineral phases.

A comparison of the stabilized and unstabilized soil samples after 1 day of curing (Fig. 9a and b) revealed no detection of new mineral phases. However, a lower-intensity peak in calcite indicated the dissolution of some mineral phases, potentially leading to the formation of a gel [41]. The calcium-aluminium-silicate hydrate (C-A-S-H) phase, a new mineralogical phase, was detected in the stabilized soil after 14, 28, and 365 days of curing (Fig. 9c, d, and 9e). These results align with previous studies, confirming that the hydration products of lime-stabilized soil begin to develop after at least 14 days [42].

The formation of C-A-S-H gel is critical to the observed improvements in soil strength and durability. This process begins with the dissociation of lime into Ca^2^⁺ and OH⁻ ions, creating an alkaline environment. The subsequent dissolution of soil minerals, specifically silica (SiO₂) and alumina (Al₂O₃), results in the release of reactive species such as [H₃SiO₄]⁻ and [H₃AlO₄]^2^⁻. These react with calcium ions to form C-A-S-H gel, as illustrated by the following chemical reactions:

Dissociation of lime:

(1) Ca(OH)_2_→Ca^2+^+2OH^−^

Dissolution of soil silica and alumina:

(2) SiO_2_+2OH^−^→[H_3_SiO_4_]^−^

(3) Al_2_O_3_+3OH^−^→2[H_3_AlO_4_]^2−^

Formation of C-A-S-H:

(4) [H_3_SiO_4_]^−^+[H_3_AlO_4_]^2−^+Ca^2+^→C−A−S−H

These reactions produce a gel that binds soil particles, increases density, and improves load-bearing capacity. Over time, the C-A-S-H phases also undergo carbonation upon exposure to atmospheric CO₂, forming calcium carbonate (CaCO₃), which further enhances soil stability:

(5) C−A−S−H + CO_2_→CaCO_3_+Silica gel

In this study, lime acted as a precursor of silica and alumina, which are crucial for producing alkali-activated cementitious materials (AACM). The primary product of AACM activation is the amorphous C-A-S-H gel, which significantly increases the density of the AACM structure [43]. This gel enhances the strength of AACM by refining pore sizes, leading to a more compact soil structure.

The increased soil strength observed with lime stabilization is due to the formation of **C-A-S-H**, as revealed by XRD analysis. This compound is known for its cementitious properties, which enhance soil cohesion and durability [44]. The notable increase in UCS is consistent with the findings of [45], who reported similar improvements in clay soils stabilized with silica-based additives. These findings support the effectiveness of lime as a stabilizer and its potential for sustainable remediation of oil-contaminated soils.

#### 4.6.2 FE-SEM and EDS analysis

**Fig. 10:**
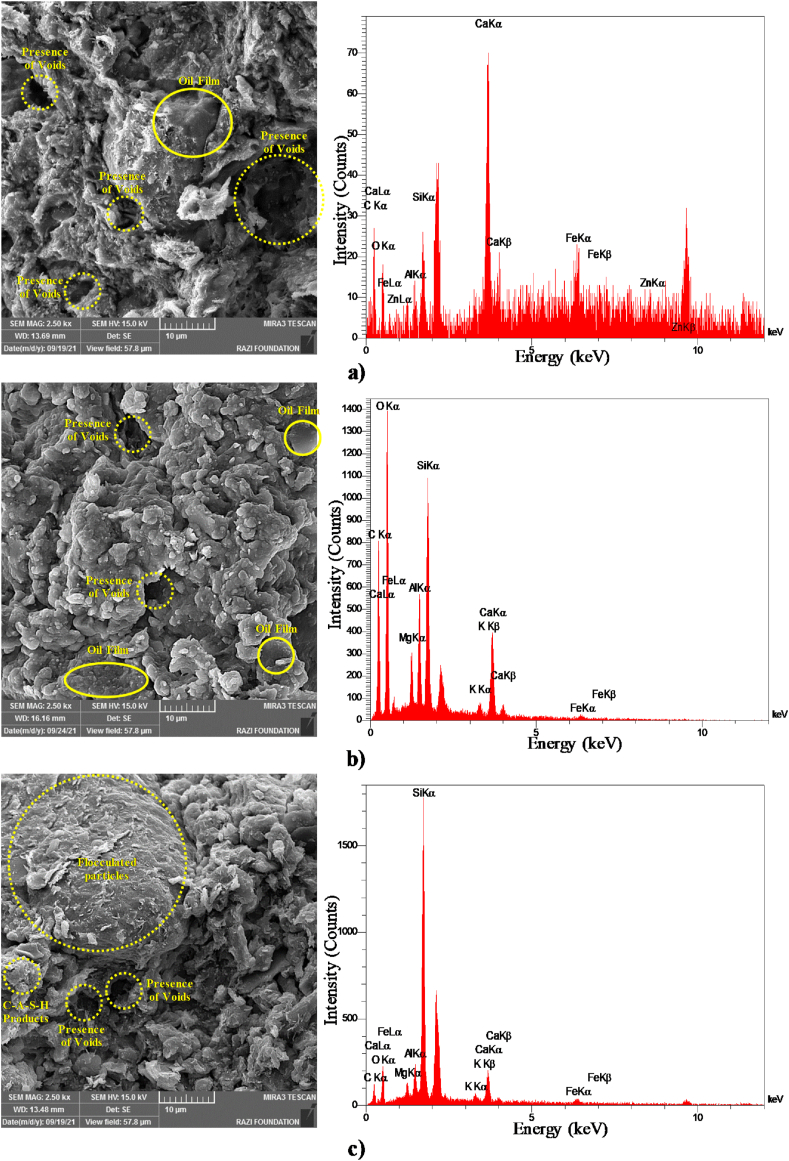

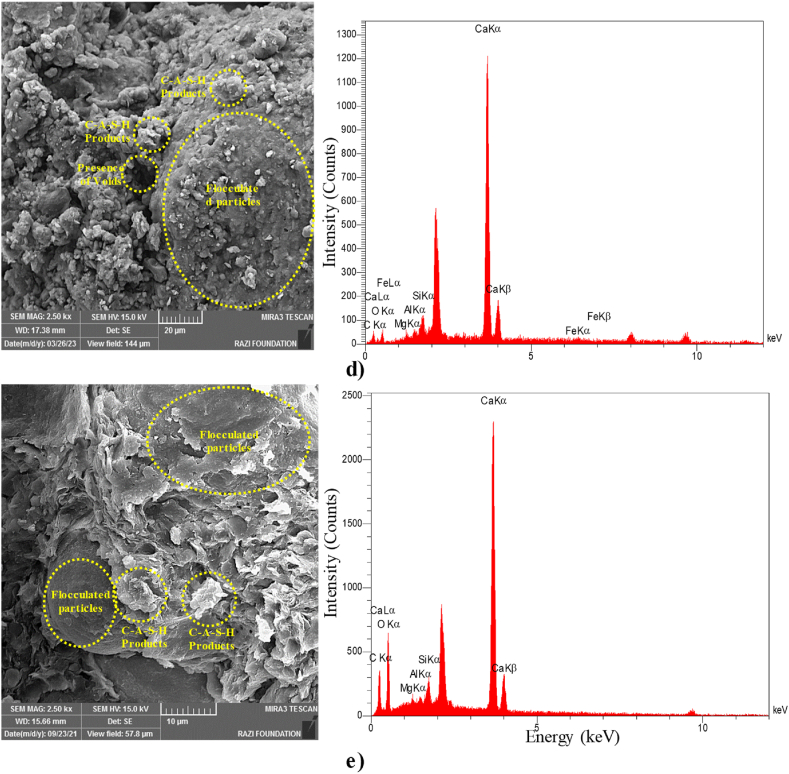
FE-SEM images and EDS spectrum of a) 10%Oil 0 % Lime Curing 28 days b) 10%Oil 6 % Lime Curing 1 day c) 10%Oil 6 % Lime Curing 14 days d) 10%Oil 6 % Lime Curing 28 days e) 10%Oil 6 % Lime Curing 365 days.

**FE-SEM** micrographs of lime-stabilized and unstabilized clayey soil are presented in Fig. 10. Additionally, EDS patterns were utilized to monitor the chemical composition changes at selected locations in the soil after stabilization with 6 % lime. High-resolution FE-SEM micrographs (2500× magnification) are provided for all samples for a more detailed comparison.

As shown in Fig. 10a, an oil film is observed, coating the soil particles. EDS results revealed the presence of calcium (Ca), aluminum (Al), silicon (Si), iron (Fe), magnesium (Mg), potassium (K), carbon (C), and sodium (Na) in the oil-contaminated sample. Fig. 10b highlights large pore spaces within the aggregates of the stabilized clayey soil after one day of treatment. This observation is consistent with Fig. 10c and d, where the 6 % lime-stabilized clayey soil contains a coherent matrix of soil particles, displaying a flocculated system with some particles covered by C-A-S-H products. In Fig. 10e, the 6 % lime-stabilized clayey soil demonstrates a robust and coherent matrix of soil particles adhered by C-A-S-H products, confirming its high strength.

Integrated with the FE-SEM examinations, EDS provides compositional variations and morphological perspectives of the tested samples’ elemental concentrations. Fig. 10 presents the EDS results for both stabilized and unstabilized mixtures. Stabilized samples displayed higher Ca content, while unstabilized samples showed negligible Ca content, confirming calcite formation as C-A-S-H.

EDS tests were conducted on oil-contaminated soil samples at various curing ages, and the compositions of C-A-S-H products were summarized. The variation of the calcium-silicon ratio (Ca/Si) and aluminum-calcium ratio (Al/Ca) concerning curing age is presented in Table 5. Notably, the Ca/Si and Al/Ca ratio trends are consistent with the Unconfined Compressive Strength (UCS) results (Fig. 4). The maximum Ca/Si ratio (2.93) and Al/Ca ratio (1.72) were recorded at 365 days, corresponding to a UCS of 3508 kPa for the 10 % oil-contaminated soil with 6 % lime stabilization.

**Table 5:** EDS analysis results for 10 % oil-contaminated soil with 6 % lime at different curing time.

Sample Name	Sample condition	Al: Ca ratio	Ca: Si ratio
10 % oil-contaminated soil with 6 % lime	1 day curing	1.23	1.95
	14 days curing	1.57	2.16
	28 days curing	1.61	2.70
	365 days curing	1.72	2.93

## 5 Conclusions

This study demonstrates the effectiveness of lime stabilization in improving the strength and durability of oil-contaminated soils, providing a sustainable solution for transportation infrastructure. Through comprehensive laboratory tests, it was found that lime treatment significantly enhances soil stability, making it a viable method for managing contaminated soils. This method supports constructing and maintaining durable infrastructure while promoting environmental sustainability by reducing the need for new materials.

Adding oil to the soil resulted in a decrease in maximum dry unit weight (MDU) and OMC, likely due to the formation of a thick oil coating around soil particles, hindering inter-particle contact and increasing slippage. It was also observed that adding lime to oil-contaminated soil leads to an increase in MDU and a decrease in OMC compared to non-contaminated soil.

The maximum UCS values were observed in soils contaminated with 7 % and 10 % oil and stabilized with 6 % lime. After one year of curing, soils stabilized with 6 % lime showed significant strength gains, with UCS increasing by 6–9 times compared to non-contaminated soils. The performance was notably better for soils contaminated with 4 % and 7 % oil than those with 10 % oil contamination.

Durability tests revealed that lime stabilization significantly improves resistance to wet-dry cycles. Soils stabilized with 6 % lime exhibited UCS improvements up to 9 times compared to unstabilized samples. Additionally, CBR tests showed that adding 6 % lime to oil-contaminated clay resulted in the most significant improvement in load-bearing capacity, with gains of up to 454 % after 28 days of curing. A high-intensity C-A-S-H phase in stabilized soil was detected after 14, 28, and 365 days, confirming its highly crystalline nature.

Lime stabilization mitigates the vulnerability of oil-contaminated soils to environmental degradation and transforms them into durable, eco-friendly construction materials. This research addresses the critical challenge of soil disposal and promotes a circular economy approach by repurposing contaminated soils, thereby reducing the need for virgin construction materials. This approach significantly lowers the ecological footprint of construction projects, contributing to sustainable resource management and advancing the transition towards more sustainable production and consumption systems.

Future research should explore lime stabilization with various contaminants across different soil types and conduct long-term field studies under diverse environmental conditions. This approach will provide deeper insights into the effectiveness and adaptability of lime stabilization in broader environmental and geotechnical applications.

## CRediT authorship contribution statement

**Hadis Nasiri:** Writing – original draft, Methodology, Investigation, Data curation. **Navid Khayat:** Writing – review & editing, Supervision, Methodology, Conceptualization. **Ahad Nazarpour:** Writing – review & editing.

## Data availability

The data that support the findings of this study are available from the corresponding author, upon reasonable request.

## Declaration of competing interest

The authors declare that they have no known competing financial interests or personal relationships that could have appeared to influence the work reported in this paper.
